# Angiopoietin-1 Requires Oxidant Signaling through p47phox to Promote Endothelial Barrier Defense

**DOI:** 10.1371/journal.pone.0119577

**Published:** 2015-03-11

**Authors:** Chandra C. Ghosh, Aditi Mukherjee, Sascha David, Katelyn E. Milam, Jon T. Hunter, Samir M. Parikh

**Affiliations:** 1 Center for Vascular Biology Research and Division of Nephrology, Beth Israel Deaconess Medical Center and Harvard Medical School, Boston, MA, United States of America; 2 Department of Nephrology and Hypertension, Hannover Medical School, Hannover, Germany; University of Illinois at Chicago, UNITED STATES

## Abstract

**Background:**

Reactive oxygen species (ROS) are largely considered to be pathogenic to normal endothelial function in disease states such as sepsis. We hypothesized that Angiopoietin-1 (Angpt-1), an endogenous agonist of the endothelial-specific receptor, Tie-2, promotes barrier defense by activating NADPH oxidase (NOX) signaling.

**Methods and Findings:**

Using primary human microvascular endothelial cells (HMVECs), we found that Angpt-1 stimulation induces phosphorylation of p47phox and a brief oxidative burst that is lost when chemical inhibitors of NOX activity or siRNA against the NOX component p47phox were applied. As a result, there was attenuated ROS activity, disrupted junctional contacts, enhanced actin stress fiber accumulation, and induced gap formation between confluent HMVECs. All of these changes were associated with weakened barrier function. The ability of Angpt-1 to prevent identical changes induced by inflammatory permeability mediators, thrombin and lipopolysaccharides (LPS), was abrogated by p47phox knockdown. P47phox was required for Angpt-1 to activate Rac1 and inhibit mediator-induced activation of the small GTPase RhoA. Finally, Angpt-1 gene transfer prevented vascular leakage in wildtype mice exposed to systemically administered LPS, but not in p47phox knock out (p47^−/−^) littermates.

**Conclusions:**

These results suggest an essential role for NOX signaling in Angpt-1-mediated endothelial barrier defense against mediators of systemic inflammation. More broadly, oxidants generated for signal transduction may have a barrier-promoting role in vascular endothelium.

## INTRODUCTION

Angiopoietin-1 (Angpt-1) stimulates Tie-2, a receptor tyrosine kinase whose expression is largely limited to the endothelium. Knockout mice for either ligand or receptor are embryonically lethal with a gross defect in vascular stabilization during developmental angiogenesis [1,2]. Based on data demonstrating persistent Tie-2 activation in adult blood vessels [3], Angpt-1 was subsequently shown to promote barrier defense in the mature, non-angiogenic vasculature against VEGF, serotonin, and mustard oil [4,5]. Using sepsis as a model condition for systemic vascular leakage, we have shown that Angpt-1 gene transfer or an Angpt-1-mimetic peptide prevents vascular leakage and improves survival in a Tie-2-dependent fashion [6,7]. Given the potential for therapeutic translation and the broad-ranging barrier defense effect against these diverse permeability mediators, insights into Angpt-1’s mechanism of action are critical.

Angpt-1 promotes endothelial cell (EC) spreading and enhanced cell-cell contacts by coordinately signaling Rho family GTPases that, in turn, regulate cytoskeletal and junctional effector proteins. Angpt-1 stimulates Rac1, which then inhibits RhoA [8–10]. When the ability of Angpt-1 to suppress RhoA is artificially blocked, Angpt-1 can no longer counteract in vitro barrier dysfunction or in vivo vascular leakage mediated by the classic RhoA activator lipopolysaccharides (LPS) [9]. The importance of endothelial RhoA activation for LPS-induced leakage in vivo has been further illustrated by an independent work showing that inhibition of the RhoA effector protein, endothelial cell myosin light chain kinase (EC-MLCK), also counteracts vascular leakage in mice following LPS challenge [11]. Therefore, addressing how Angpt-1 inhibits RhoA is critical to understanding its barrier defense mechanism.

The NADPH oxidase complex is comprised of membrane-bound flavocytochrome b558 (composed of p22phox and gp91phox/NOX2) and cytosolic regulatory subunits ofp47phox, p40phox, p67phox and Rac1 or Rac2 [12]. The precise role of Angpt-1 in generation of endothelial ROS via NADPH oxidase and thereby Rac1 activation/RhoA inhibition is not clearly established. It is believed that phosphorylation of p47phox is required for receptor-mediated NOX2 activation and intercellular ROS generation[12]. We hypothesized that Angpt-1 mediates p47-dependent NADPH oxidase (NOX) activity which may contribute to Angpt-1-mediated RhoA suppression and barrier defense in microvascular endothelium. Previous work has suggested a role for NOX-derived oxidants in the angiogenic actions of Angpt-1 [11,13,14], but in vascular leakage related to systemic inflammation, oxidants are widely considered to be mediators of leak rather than signaling components of the barrier defense response [15].

To test this hypothesis, we first confirmed that Angpt-1 application to HMVECs induced a NOX-dependent oxidative burst. Next, we found that the chemical inhibition of NOX2 and p47phox component was sufficient to block the Angpt-1-mediated oxidative burst. Finally, we evaluated endothelial architecture, barrier function, and in vivo vascular leakage in a series of experiments using Angpt-1, LPS, thrombin, NOX-2 inhibitor and genetic manipulation of p47phox. Our results suggest that a NOX-dependent oxidative burst not only follows Angpt-1 stimulation, but is critical to the suppression of RhoA and barrier defense against inflammatory permeability mediators.

## MATERIALS AND METHODS

### Antibodies and Reagents

Antibodies used for immunoblotting and immunocyto/-histochemistry were purchased from the following manufacturers: p47phox and actin (C-11) from Santa Cruz (Santa Cruz, CA), phospho-p47^s304^ from Abcam (Cambridge, MA), Tie2 from Millipore (Danvers,MA), VE-cadherin from BD Bioscience (San Jose, CA), and anti Rac1 and anti RhoA from Cytoskeleton (Denver, CO).CM-H_2_DCFDA, Phalloidin, DAPI (4′,6-diamidino-2-phenylindole),and fluorophore- or horseradish peroxidase (HRP)-conjugated secondary antibodies were purchased from Life Technologies (Carlsbad, CA).Thrombin was procured from Calbiochem (San Diego, CA). LPS (O111:B4)/LBP/CD14, Phospho-Tie2^Y992^, recombinant Angpt-1 were procured from R&D Biosystems (Rochester, MN). The adenovirus expressing Angpt-1 and GFP were obtained from Qbiogene Inc (Carlsbad, CA).Other reagents were purchased from Sigma-Aldrich (St. Louis, MO).

### Cell Culture and Reagents

Passage 4–6 human microvascular endothelial cells (HMVECs) from dermis (Lonza) were cultured on collagen I (Advanced BioMatrix Inc.) in EBM-2 media (Lonza) supplemented with 5% FBS and growth factors. Apocynin (Apo), siRNAs, and Lipofectin were purchased from Life Technologies. Hyper-3 (pC1-HyPer-3) was obtained from Addgene (Cambridge, MA)[16] and was transfected using X-fect transfection kit from Clontech (Mountain View, CA) as per manufacturer's instruction.

### Mouse Study

This study was carried out in strict accordance with the recommendations in the Guide for the Care and Use of Laboratory Animals of the National Institutes of Health. The Institutional Animal Care and Use Committee at Beth Israel Deaconess Medical Center (Animal Welfare Assurance Number: A3153-01) approved this protocol. Male p47phox knockout mice and their wildtype littermates on C57BL6/J background were purchased form Jackson Labs (Bar Harbor, Maine).

### Reactive Oxygen Species (ROS) measurement

#### DCFDA studies

Intracellular ROS were determined based on the principle of oxidative conversion of cell permeable chloromethyl-2′,7′-dichlorodihydrofluorescein diacetate to fluorescent dichlorofluorescein (DCF) as mentioned elsewhere[13]. Briefly, confluent HMVECs (P5) were serum starved with 1% FBS overnight, incubated with 10 μM CM-H_2_DCFDA in PBS for 30 minutes before stimulation with Angpt-1 (300ng/ml). DCF fluorescence was measured over the whole field of vision using a Zeiss fluorescence microscope connected to an imaging system. Ten high-power images per experiment were obtained for later planimetric quantification using a single threshold value for all images in Adobe Photoshop as described previously [17].

#### Live cell imaging to measure ROS

To overcome the non-specificity of the chemical probes, Hyper-3, a plasmid based biosensor can efficiently measure the generation of intercellular ROS in real time [16].Fifty to sixty percent confluent of HMVECs were grown in Lab-TekII chambered plates (Thermo Scientific, Rochester, NY) and were transfected with Hyper-3 plasmid using X-fect (Clontech) as per the manufacturer's protocol. After 48 hours of transfection, cells were serum starved for 2 hours before addition of chemicals such as VAS2870 (endothelial NOX2 and NOX4 inhibitor)[18] and Apo. Thirty minutes after the addition of the chemicals cells were treated with Angpt-1 and H_2_O_2_. The images were taken in Zeiss LSM510 META live cell confocal microscope confocal system at x20 with as described earlier with single wave length monitoring [19]. Of note, all images were obtained with the same laser power, gain, and offset conditions. The intensity of the cells before and after treatment was measured by ImageJ (NIH, Baltimore, MD) and the fold change in the intensity of the same cell was considered for calculation.

### RNA interference

The siRNA-mediated interference in HMVECs were achieved as described previously with small modifications[8]. Briefly, HMVECs were grown to 70–80% confluence in 6-cm plates and transfected with 5, 20nMp47phoxsiRNA against p47phox (5-GGUCAUUCACAAGCUCCUGtt-3, Ambion, Austin, TX), or scrambled siRNA(Scramsi) in Opti-MEM containing 10μg/ml Lipofectin as per manufacturer's protocol (Life Technologies, Carlsbad, CA). Cells were harvested after 48 hours of transfection.For ECIS experiments we used HyPerFect (Qiagen, Valencia, CA) with a fast forward protocol recommended by the manufacturer.

### Immunocytochemistry

HMVECs were grown to confluence on glass cover-slips coated with collagen type I. After starvation, the cells were treated with LBP (100ng/mL) and CD14 (10ng/mL) for 30 minutes before treating with LPS (100ng/ml) in presence or absence of Angpt-1(300ng/ml) for another 30 minutes. Cells were fixed for 10 minutes with 2.5% para-formaldehyde and permeabilized for 5 minutes with 0.2% Triton X-100 in PBS. Cells were blocked overnight at 4°C with a blocking buffer (1% BSA, 0.1% Triton X-100, 0.1% sodium azide), then incubated for 12 hours with primary antibody, serial washes in PBS, then a 60 minute incubation with secondary Alexa-fluor-antibody and phalloidin. The coverslips were mounted by using ProLong Gold/DAPI. All images were taken by a Zeiss LSM510 META confocal system at X63. Of note, all images were obtained with the same laser power, gain, and offset conditions.

### RhoA/Rac1GTP measurements

Rho-GTP was measured in 100% confluent HMVECs. Cells were treated with Apo (650 uM), Angpt-1 (300 ng/mL), and with or without Thrombin (1 U/ml) for 15 minutes to activate RhoA. Protein lysates were collected, and activated GTP-bound RhoA was analyzed with a GLISA activation assay kit (Cytoskeleton, Denver, CO) according to the manufacturer's instructions.

### Western Blot Analysis

Cells or organ lysates were prepared by homogenization in ice-cold RIPA buffer (Boston BioProducts, Boston, MA) supplemented with protease inhibitors (Roche Diagnostics, Indianapolis, IN), 1 mM EDTA, 1 mM Na_3_VO_4_, and 1 mMNaF. Lysates were sonicated and centrifuged at 8,000 *g* for 10 minutes at 4°C, and supernatants were collected. Electrophoresis, transfer, immunoblotting, detection, and image acquisition were performed as previously described [20,21].

### Trans-endothelial electrical resistance

HMVECs were grown to confluence in polycarbonate wells containing evaporated gold microelectrodes in series with a large gold counter connected to a phase-sensitive lock-in amplifier as described previously [22–24]. To silence p47phox we used 20nM of p47phoxsiRNA and Fast Forward protocol as per manufacturer's direction (Qiagen)[25]. Twenty-four hours after of transfection, the confluency of cells were evaluated and base line resistance was measured in an electrical cell-substrate impedance sensing system (ECIS-1600) (Applied Biophysics, Troy, NY) as described before [6,8,26].When the cells reached the resistance more than 1500 ohm generally 36–48 hours of post transfection, cells were treated with Angpt-1, Thrombin, LPS, LBP, and CD14.

Measurements of TER were performed in real-time and values from each microelectrode were pooled at discrete time points and either plotted versus time as the mean ± SEM or reported as bar graphs at the time-point of maximal response to a given stimulus as described elsewhere in detail [27].

### Mouse Evans blue (EB) permeability assay in lungs

To measure the LPS induced vascular leak we injected LPS (15mg/kg body weight) to 8–12 week-old male mice. Viral particles (2 × 10^10^) of Ad-Angpt-1 or control adenovirus (Ad-CMV-GFP) were administered by tail vein 48 hours before LPS administration. Sixteen hours after LPS injection, animals were deeply anesthetized with inhaled isoflurane and EB dye was injected intravenously as previously described [6,28,29]. The following formula was used to correct the optical densities (shown as E_(wavelength)_) for contamination with heme pigments:

*E*620(*corrected*) = *E*620(*raw*)−(1.426 *x E*740(*raw*) + 0.03)

### Statistical analysis

Data are presented as mean ± SEM unless otherwise noted. For statistical comparisons between two groups, we used unpaired, two-tailed Student's *t*-test. For comparisons of 3 or more groups, we used One-way analysis of variance (ANOVA) with Bonferroni's multiple comparison post hoc test. Differences of p < 0.05 were considered significant. The results were analyzed in GraphPadPrism5.0 (San Diego, CA).

## RESULTS

### Angpt-1 induces a p47phox-dependent oxidative burst in endothelium

**Fig 1:**
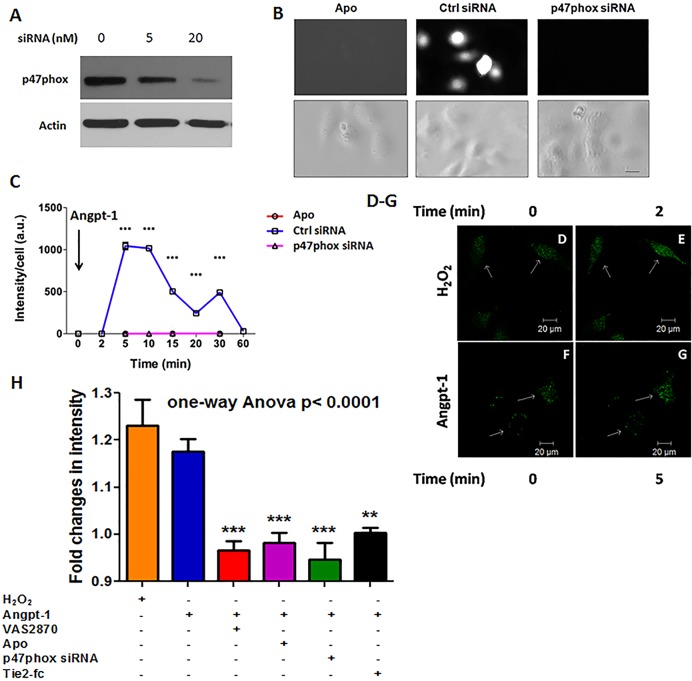
Angpt-1 induces a p47phox-dependent oxidative burst in endothelium. (**A**) Titration and validation of p47phox siRNA delivery into HMVECs. (**B**) Example epifluorescence (excitation 495 nm: emission 520 nm) and corresponding bright field images of individual HMVECs loaded with CM-H_2_DCFDA, pre-treated with Apo (650 μM), control siRNA, or p47phox siRNA, and then treated with Angpt-1 (300 ng/ml). Images shown were taken 5 minutes after Angpt-1 addition. Scale bar 10 μm. (**C**) Planimetric quantification of CM-H_2_DCFDA fluorescence from above conditions (n = 3–5 experiments per condition). ***p<0.001 compared to p47phox siRNA+Angpt-1. **(D-G)** Cells transfected with Hyper-3 for 48 hours, underwent serum starvation for 2 hours, were treated with 10 μM H_2_O_2_ or Angpt-1 (300 ng/ml), and changes in fluorescence intensity were measured by live cell imaging microscopy. Representative images after 2 minutes of H_2_O_2_ **(D-E)** and 5 minutes with Angpt-1 **(F-G)** are shown. **(H)** Hyper-3 transfected HMVECs were, treated with chemicals (Apo and VAS 2870), siRNA p47phox or Tie2-Fc (500 ng/ml) before addition of Angpt-1 (300 ng/ml). Results were analyzed by one-way ANOVA followed by post-hoc corrections for multiple comparisons. ***p<0.001,***p<0.01 relative to Angpt-1 alone.

Given the role of NOXs in regulating GTPases and our previous findings in support of a GTPase mechanism of Angpt-1 barrier defense [8,9], we hypothesized that NOXs may be involved in Angpt-1-mediated barrier defense. We focused on p47phox based upon a large body of literature describing its functions in the endothelium (summarized in [30]. We first validated a siRNA against p47phox in HMVECs (**Fig. 1A**). We then used the cell-permeable reactive oxygen species detector dye, DCFDA, to follow the oxidative burst induced by Angpt-1 as previously reported [11,13,14]. We found that the chemical NOX inhibitor, Apo, or p47phox siRNA was sufficient to abrogate this burst (**Fig. 1B,C**). To overcome the non-specificity of the chemical redox probe, we used genetically defined redox probe-plasmid, Hyper-3[16,19].Using time-lapse imaging, we observed that H_2_O_2_ as well as Angpt-1 generate intracellular ROS **(Fig. 1D-G)**.The effect of Angpt-1 was significantly reducedwhen Hyper-3 transfected cellswere pretreated with VAS2870, Apo, or p47phox siRNA (**Fig. 1H**).We also explored whether Angpt-1 phosphorylates p47phox and may alter the localization of p47phox from cytosol to membrane to augment ROS production. We found that Angpt-1 induces phosphorylation of p47phox at serine-304, an important regulatory site for p47phox (**S1 Fig.**).

### Angpt-1 requires NOX activity to inhibit thrombin-induced RhoA activation

**Fig 2:**
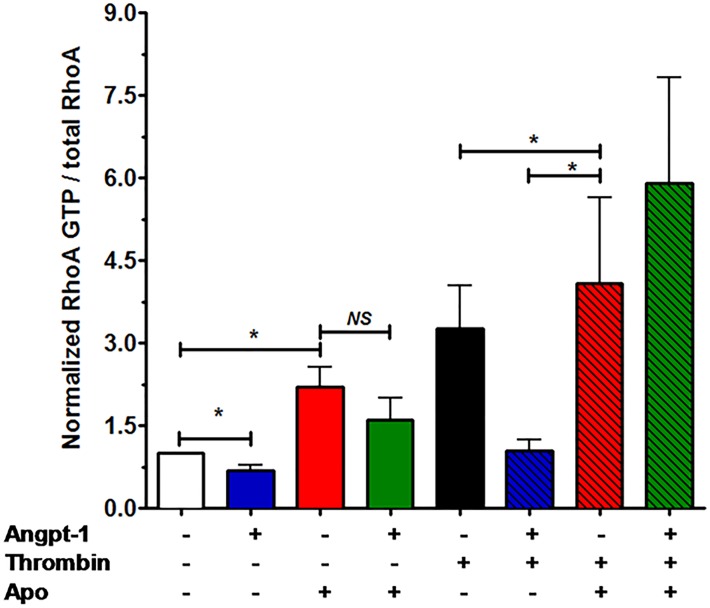
Angpt-1 requires NOX activity to inhibit thrombin-induced RhoA activation. Confluent HMVECs were treated with vehicle (NT), Angpt-1 (300 ng/ml), Apo (650 μM), and/or thrombin (1 U/ml). Cells were lysed in the manufacturer-provided buffer 15 minutes after treatments for RhoA G-LISA, a pulldown method for RhoA-GTP detection. The results were then normalized to total RhoA, as determined by densitometry of Western blots (n = 4–6 experiments per condition). *p< 0.05.

We have previously described that Angpt-1 activates Rac1, which in turn, inhibits RhoA. this mechanism is required for Angpt-1-mediated barrier defense [8,9]. We used a canonical stimulator of RhoA, thrombin, to test whether NOX activity was necessary for Angpt-1 to inhibit thrombin-induced RhoA activation (measured as the GTP-bound form of RhoA). Our results showed that Angpt-1 inhibits both basal and thrombin-induced RhoA activation (**Fig. 2**). In unstimulated HMVECs, Apo induced RhoA activation, suggesting basal NOX activity is unable to prevent RhoA activity; therefore, Angpt-1 could no longer inhibit RhoA in this situation. These results demonstrate the reliance of Angpt-1 on NOXs to prevent mediator-induced RhoA activation.

### p47phox enables Angpt-1 to counteract cellular structural rearrangements induced by thrombin

**Fig 3:**
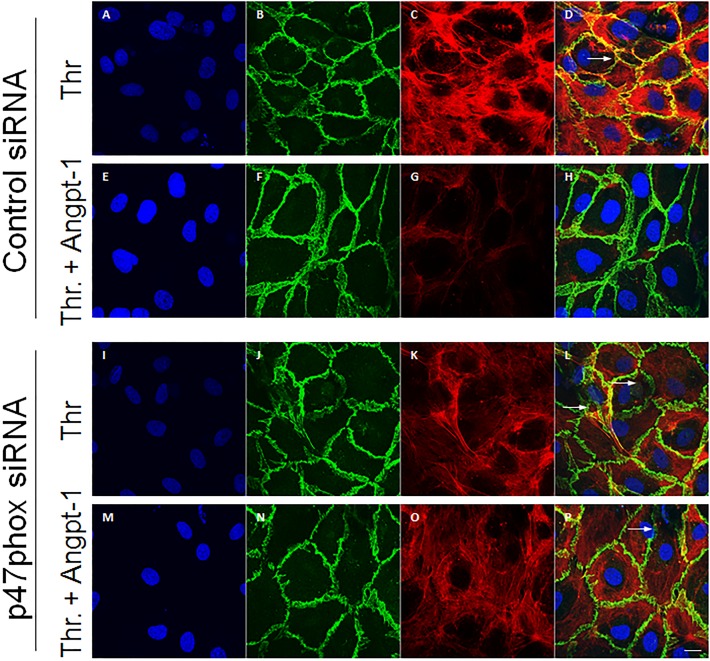
p47phox enables Angpt-1 to counteract cellular structural rearrangements induced by thrombin. (**A-H**) Confluent HMVECs were treated with control siRNA, thrombin (1 U/ml, **A-D**), or the combination of Angpt-1 (300 ng/ml) plus thrombin (**E-H**). After 15 minutes, cells were fixed, permeabilized, and stained for nuclei (blue, DAPI), VE-cadherin (green), or F-actin (red). White arrows indicate paracellular gaps. (**I-P**) Repeat of the above experiments, replacing control siRNA with p47phox siRNA. Representative of n = 3–4 experiments per condition. Scale bar 10 μm.

The balance of intracellular GTPases dictates the cytoskeletal architecture of endothelial cells, which in turn may regulate permeability [31]. We predicted that actin stress fibers and paracellular gaps induced by thrombin and prevented by Angpt-1 would involve p47phox. To test this, we used control or p47phox siRNA on HMVECs. While p47phox knockdown alone had no discernible effect on stress fibers or gaps (data not shown), the ability of Angpt-1 to counteract the cytoskeletal and junctional effects of thrombin was lost upon p47phox reduction (**Fig. 3A-P**).

### P47phox is required for Angpt-1-mediated barrier defense against thrombin and LPS

The electrical resistance of a confluent cellular monolayer provides a sensitive measure of real-time changes in monolayer permeability. Thrombin has a well-described permeability effect on ECs in this assay that relies on activated RhoA[32]. Gram-negative lipopolysaccharide (LPS) is a medically important mediator of vascular leakage in severe infectious syndromes[33]. LPS induces endothelial barrier dysfunction that is both RhoA-dependent and preventable by Angpt-1 application [6,9]. To explore whether Angpt-1-mediated barrier defense against LPS also requires NOX signaling, we first studied the HMVEC cytoskeleton and junctions using Apo. The ability of Angpt-1 to prevent LPS-induced structural changes was lost upon Apo addition (**S2 Fig.**).

**Fig 4:**
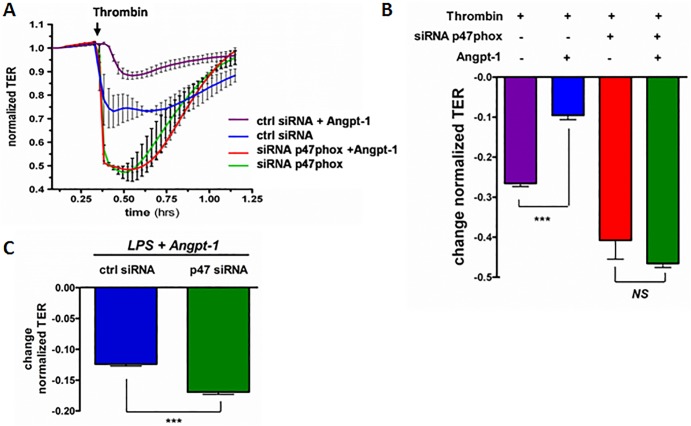
p47phox is required for Angpt-1-mediated barrier defense against thrombin. (**A**) Transendothelial resistance assay (TER) of confluent HMVECs treated with thrombin (1 U/ml), with p47phox or control siRNA, and with and without Angpt-1 (300 ng/ml). n = 4 experiments per condition. To enable comparisons between conditions, the baseline absolute resistance of each well was used to normalize subsequent readings for the respective well. (**B**) Data from (**A**) quantified as the change in normalized resistance 30 minutes after thrombin addition. ***p<0.001. **(C)** Transendothelial resistance assay (TER) of control- vs. p47phox-siRNA-treated HMVECs to which LPS (10 ng/ml) and Angpt-1 (300 ng/ml) were applied. The change was recorded 1 hour after LPS and Angpt-1 addition (n = 3 experiments per condition). ***p< 0.001, n = 4 experiments per condition.

Based on the ability of Angpt-1 to counteract thrombin-induced RhoA activation in a p47phox-dependent fashion (**Fig. 3**), we asked whether Angpt-1 could counteract thrombin-induced barrier dysfunction, and if so, whether this form of barrier defense required p47phox. Compared to control siRNA, p47phox silencing had no effect on basal barrier function without the presence of thrombin (**Fig. 4A**, left of thrombin arrow). Upon addition of thrombin, p47phox-siRNA HMVECs responded with a greater fall in resistance than control-siRNA HMVECs. Angpt-1 substantially attenuated the thrombin-induced fall in resistance of control siRNA-treated HMVECs. This protective effect was lost in p47phox-siRNA HMVECs (**Fig. 4A,B**). These results show that reduction of p47phox exacerbates barrier dysfunction induced by the canonical RhoA activator, thrombin, and that Angpt-1’s barrier-protective effect against thrombin relies on intact p47phox expression.

In a previous study, we observed that Tie-2 stimulation with a synthetic Angpt-1-peptide mimetic counteracted the LPS-induced fall in electrical resistance of confluent HMVECs [6]. We therefore used the same assay to test the role of p47phox. Compared to control siRNA, knockdown of p47phox reduced the resistance of confluent HMVECs treated with LPS+Angpt-1 (**Fig. 4C**). Of note, HMVEC monolayers treated with LPS alone showed a 20% drop in resistance, statistically indistinguishable from LPS+Angpt-1+p47phox siRNA (data not shown). Together with the thrombin studies in HMVECs, these data provide evidence that Angpt-1-mediated barrier defense against RhoA-dependent mediators of permeability requires p47phox.

### Angpt-1 mediated barrier defense in acute systemic inflammation requires p47phox

**Fig 5:**
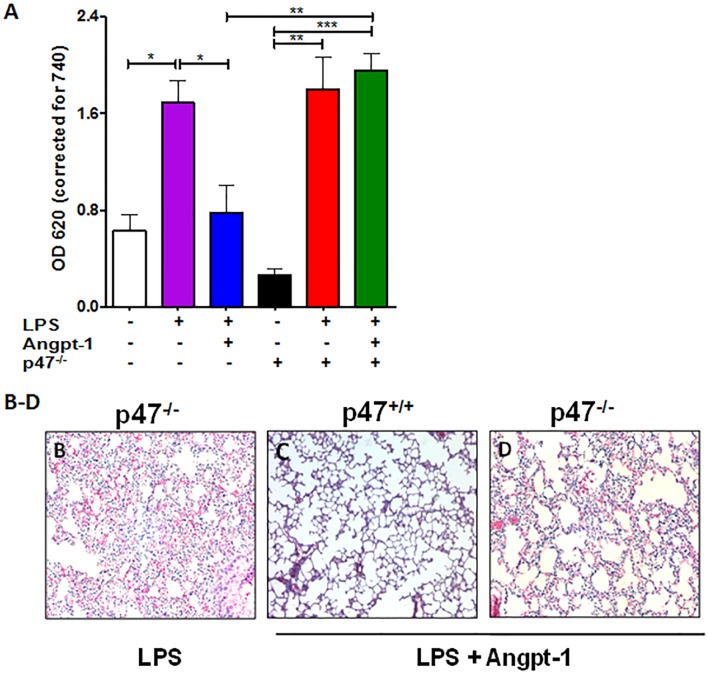
Angpt-1 mediated barrier defense in acute systemic inflammation requires p47phox. (**A**) Spectrophotometric quantification at 620 nm of intravenously injected Evans blue dye extravasation into the lungs of wildtype littermates (p47^+/+^) and p47phox KO mice (p47^−/−^) 16 hours after LPS (15 mg/kg IP) with prior control adenovirus (control) or Angpt-1 adenovirus (1 x 10^9^pfu/mouse) gene transfer. (**B-D**) Lung photomicrographs from above conditions (representative of n = 3–5 mice per condition). Scale bar 50 μm.

We and others have previously shown that Angpt-1 (or mimetics thereof) counteract critical manifestations of sepsis in experimental rodent models [6,7,9,34–37]. LPS injection in mice induces systemic inflammation that mimics features of the early cytokine storm in sepsis, such as severe vascular leakage. To evaluate the significance of the proposed p47phox-dependent barrier defense mechanism of Angpt-1 in vivo, we administered LPS with or without Angpt-1 gene transfer by adenovirus in p47^−/−^ mice and their wildtype littermates. LPS induced comparable vascular leakage in p47^−/−^ and wildtype mice. Inwildtype mice, Angpt-1 gene transfer prevented the LPS-induced increase in lung permeability (**Fig. 5A**). However, the anti-permeability effect of Angpt-1 against LPS was lost in p47^−/−^ mice. In addition, LPS functioned as a “double-edged sword” by decreasing the activation of pTie2 and reducing the level of total Tie2 protein without affecting the total protein level of Rac1 (**S3 Fig.**). As previously shown [9,35], LPS-induced cellular infiltration of the lungs was also attenuated by Angpt-1, but again, deletion of p47phox abrogated this protective effect (**Fig. 5B-D**).

## DISCUSSION

Our results show that Angiopoietin-1 counteracts endothelial barrier dysfunction induced by canonical inflammatory mediators, LPS and thrombin, by signaling through the NOX subunit p47phox. To arrive at this conclusion, we used chemical and genetic manipulations of Angpt-1-induced NOX signaling with a combination of three techniques to assess barrier modulation—immunocytochemistry of cytoskeletal and junctional proteins, the highly sensitive electrical resistance assay, and adult mice in which vascular leakage was provoked by LPS challenge. The results suggest that oxidant production is a critical component of the signal transduction cascade that enables Angpt-1 to promote endothelial barrier defense.

Previous work on the role of oxidants in endothelial barrier function has generally proposed that these molecules are mediators of increased vascular permeability, particularly since reactive oxygen species are generated during ischemia-reperfusion injury and by activated neutrophils (reviewed in [15,38]). However, experimental systems for studying oxidant-induced endothelial barrier dysfunction, such as application of H_2_O_2_ to EC monolayers, may not be an appropriate comparison for interpreting the effects we observed following Angpt-1 application. First, the oxidative burst we observed is brief compared to the sustained exposure to H_2_O_2_ necessary to provoke barrier dysfunction. Second, and perhaps more importantly, the production of a superoxide following Angpt-1 is likely restricted to signaling complexes assembled around Tie2 and NOXs. Although speculative, compartmentalization of the oxidative burst may also help explain how NOX activity is also downstream of endothelial pathways such as VEGF that are known to enhance permeability [39,40].

Several studies have reported that Angpt-1 stimulates NOX activity, implicating this pathway in the migration of ECs [11,13,14]. None, however, have asked whether NOX activity also contributes to barrier defense of confluent ECs. One potential explanation for these two apparently discrepant phenotypes observed in cell culture may relate to the experimental conditions. In sparsely cultured cells, activation of Rac1 induces dorsal ruffles and, in gradient settings, activated Rac1 accumulates at the leading edge of the migrating cell where it promotes cell spreading through lamellipodia [41]. By contrast, there is no “room” for migration among confluent cells. Nonpolar Rac1 activation in this setting may promote spreading of cells in all directions, resulting in enhanced physical overlap between adjacent cells [31]. Such a mechanism could account for the enhanced barrier function that follows Rac1 stimulation in confluent settings, while also explaining Rac1-dependent migration in sparse settings. Studies also show the importance of Rac1 and p47phox localization—for example, membrane-to-cytosol translocation may be responsible for enhanced barrier integrity [42].

Further questions are suggested by our results. First, is NOX activation a general feature of endothelial barrier defense regardless of the upstream pathway? Our data with chemical inhibition of NOX activity supports this hypothesis, but more experiments are needed. Second, global knockout mice for p47phox have impaired neutrophil function; therefore, an endothelial-specific p47phox deletion would be even more informative about the role of NOX signaling in endothelium in vivo. However, we should note that KO mice and their wildtype littermates had equivalent permeability responses to LPS. Furthermore, the highly restricted expression of Angpt-1’s receptor, Tie2, to endothelium confers additional cellular specificity to the in vivo results. Nonetheless, Angpt-1 could have acted on putative non-Tie-2 receptors [43], and extra-endothelial effects of p47phox deletion could still have factored into the observed permeability results. [8,9]. We also observed that Angpt-1 phosphorylates p47phox at S-304, one of the important sites of p47phox phosphorylation (**Fig. 2A,B**) that is not completely reversed by treating the cells with NOX inhibitor [44]. Finally, compartmentalization of the NOX oxidative burst in ECs merits further characterization.

In summary, the present work identifies p47phox-dependent NOX activity as a critical component of Angpt-1-mediated endothelial barrier defense against classic inflammatory permeability factors. These results advance our understanding of the wide-ranging barrier defense signaled by Angpt-1 and, more broadly, suggest that oxidants within ECs may have barrier-promoting properties.

## Supporting Information

S1 Fig Angpt-1 induces p47phox phosphorylation in endothelium. **(A)** Confluent HMVECs were serum-starved overnight, then treated with VAS2870 (10 μM) for 30 minutes before addition of Angpt-1 for 15 minutes. Protein lysates were resolved by Western and probed for phospho-p47phox^s304^, total p47phox. Equal loading was confirmed by detecting total Rac1. **(B)** Densitometry analysis showing the ratio of phospho p47phox^S304^/total p47phox. *p< 0.05;**p < 0.01, n = 3 per condition).
(TIF)

S2 Fig The role of p47phox in Angpt-1-mediated barrier defense extends to LPS. (**A-D**) Confluent HMVECs were treated with Apo (650 μM) 30 minutes prior to LPS (10 ng/ml) with or without Angpt-1 (300 ng/ml) for 30 minutes and then stained for VE-cadherin (green) and F-actin (red). White arrows indicate paracellular gaps. Representative of n = 3 experiments per condition. Scale bar 10 μm.
(TIF)

S3 Fig Stable Tie2-signaling in p47phox^−^^/^^−^ mice. Lung lysates of p47phox^−/−^ mice after transduction with Ad-Angpt-1 or Ad-GFP. The levels of pTie2 and pTie2 were evaluated by Western analysis.
(TIF)
