# Supplementary figures and images for: Angiopoietin-1 Requires Oxidant Signaling through p47phox to Promote Endothelial Barrier Defense
Source: PLoS One. 2015 Mar 11;10(3):e0119577. doi: 10.1371/journal.pone.0119577 (PMC4356555; doi:10.1371/journal.pone.0119577)

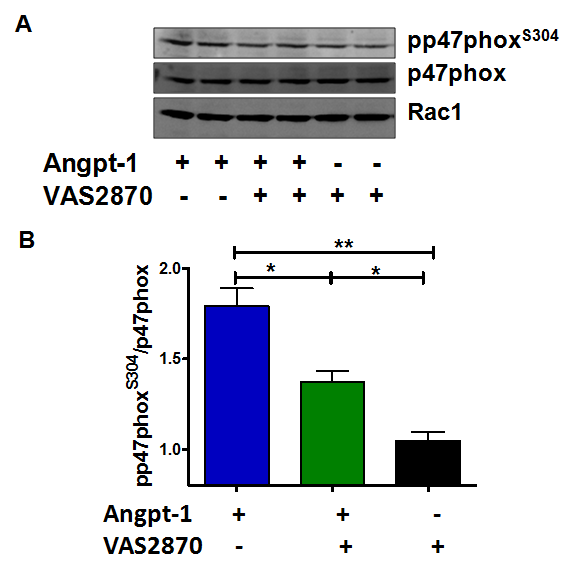

Supplement: S1 Fig — (A) Confluent HMVECs were serum-starved overnight, then treated with VAS2870 (10 μM) for 30 minutes before addition of Angpt-1 for 15 minutes. Protein lysates were resolved by Western and probed for phospho-p47phoxs304, total p47phox. Equal loading was confirmed by detecting total Rac1. (B) Densitometry analysis showing the ratio of phospho p47phoxS304/total p47phox. *p< 0.05;**p < 0.01, n = 3 per condition). (TIF) [file pone.0119577.s001.tif]

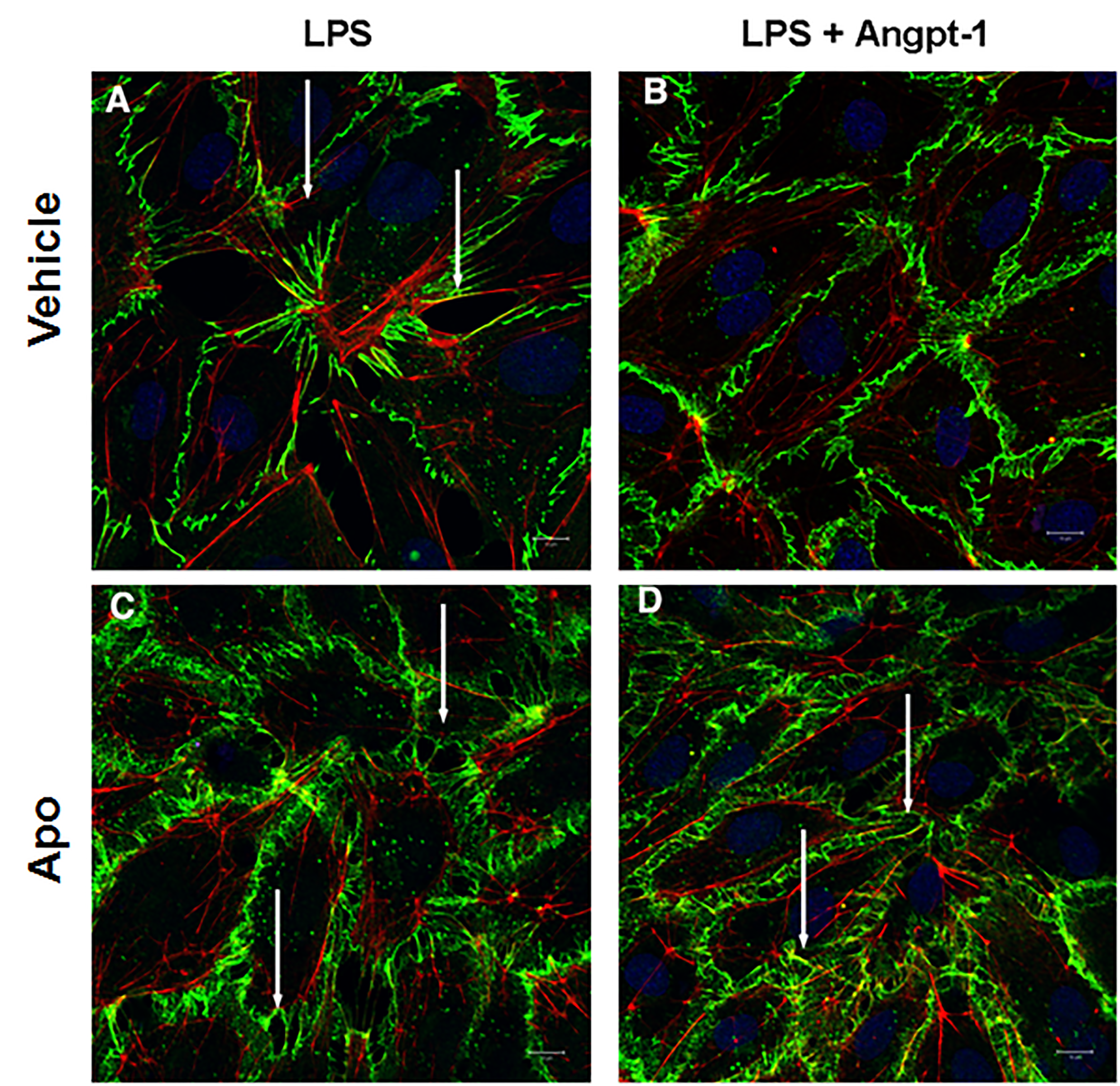

Supplement: S2 Fig — (A-D) Confluent HMVECs were treated with Apo (650 μM) 30 minutes prior to LPS (10 ng/ml) with or without Angpt-1 (300 ng/ml) for 30 minutes and then stained for VE-cadherin (green) and F-actin (red). White arrows indicate paracellular gaps. Representative of n = 3 experiments per condition. Scale bar 10 μm. (TIF) [file pone.0119577.s002.tif]

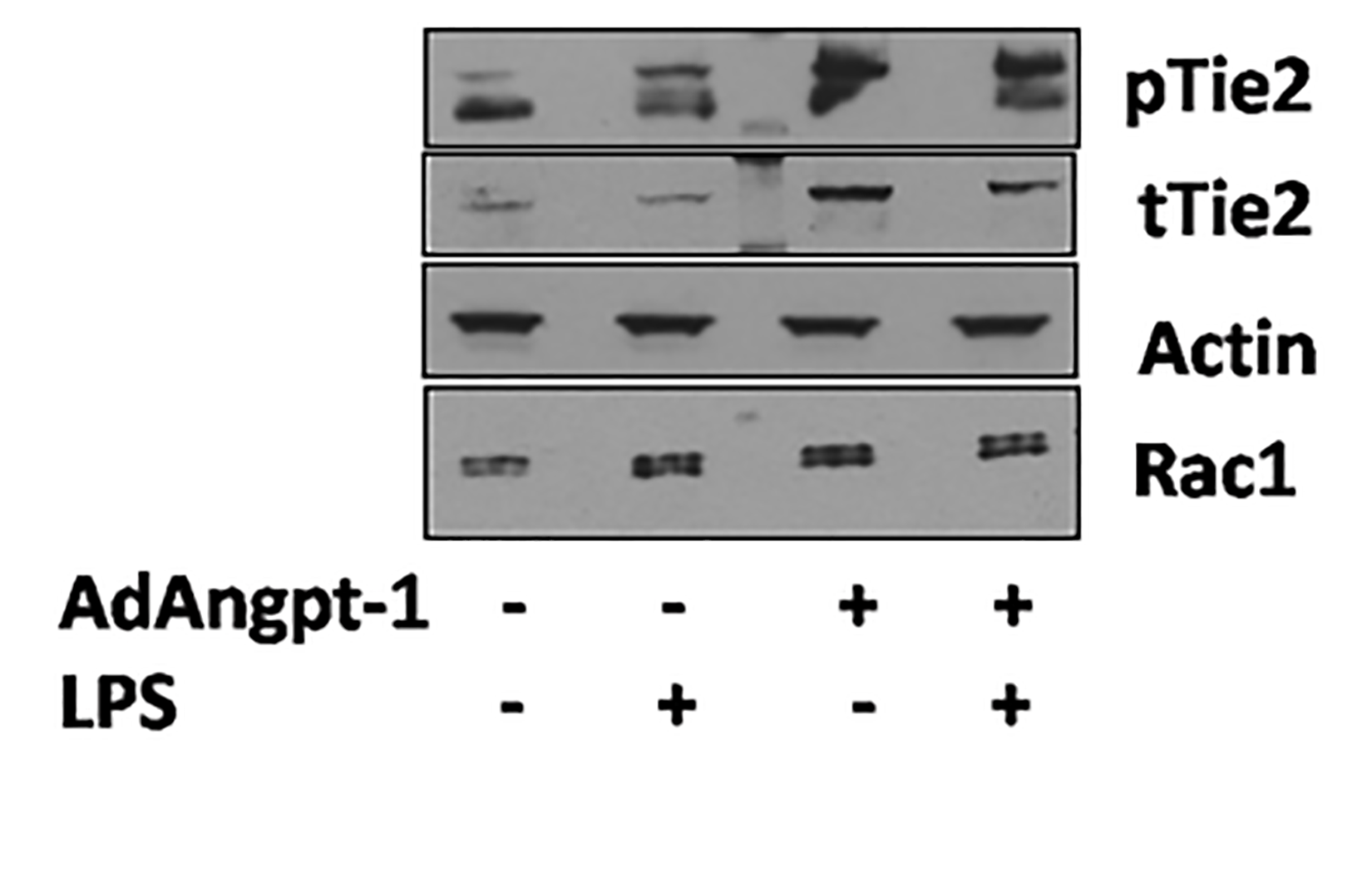

Supplement: S3 Fig — Lung lysates of p47phox−/− mice after transduction with Ad-Angpt-1 or Ad-GFP. The levels of pTie2 and pTie2 were evaluated by Western analysis. (TIF) [file pone.0119577.s003.tif]
